# Rapid Gene Turnover as a Significant Source of Genetic Variation in a Recently Seeded Population of a Healthcare-Associated Pathogen

**DOI:** 10.3389/fmicb.2017.01817

**Published:** 2017-09-20

**Authors:** Lucía Graña-Miraglia, Luis F. Lozano, Consuelo Velázquez, Patricia Volkow-Fernández, Ángeles Pérez-Oseguera, Miguel A. Cevallos, Santiago Castillo-Ramírez

**Affiliations:** ^1^Programa de Genómica Evolutiva, Centro de Ciencias Génomicas, Universidad Nacional Autónoma de México Cuernavaca, Mexico; ^2^Departamento de Enfermedades Infecciosas, Instituto Nacional de Cancerología Mexico, Mexico

**Keywords:** population genomics, microevolution, genetic variation, pathogen, phylogeography, *A. baumannii*, gene content

## Abstract

Genome sequencing has been useful to gain an understanding of bacterial evolution. It has been used for studying the phylogeography and/or the impact of mutation and recombination on bacterial populations. However, it has rarely been used to study gene turnover at microevolutionary scales. Here, we sequenced Mexican strains of the human pathogen *Acinetobacter baumannii* sampled from the same locale over a 3 year period to obtain insights into the microevolutionary dynamics of gene content variability. We found that the Mexican *A. baumannii* population was recently founded and has been emerging due to a rapid clonal expansion. Furthermore, we noticed that on average the Mexican strains differed from each other by over 300 genes and, notably, this gene content variation has accrued more frequently and faster than the accumulation of mutations. Moreover, due to its rapid pace, gene content variation reflects the phylogeny only at very short periods of time. Additionally, we found that the external branches of the phylogeny had almost 100 more genes than the internal branches. All in all, these results show that rapid gene turnover has been of paramount importance in producing genetic variation within this population and demonstrate the utility of genome sequencing to study alternative forms of genetic variation.

## Introduction

Genome sequencing of bacteria has drastically transformed our view of how bacteria change. For example, the use of genome sequencing in microbial experimental evolution has been of paramount importance to better understand the mechanisms that generate genetic variation in bacterial populations. However, these types of experiments are far from what really happens in nature—as they are simplified versions of natural populations. On the other hand, natural populations of bacterial pathogens themselves provide a copious source to address the question of how bacteria evolve. Over the last two decades genome sequencing has allowed us to better understand not only the microevolution but also the epidemiology of many bacterial pathogens. For instance, this tool has been used to study the phylogeography and population structure of several human and animal bacterial pathogens (Brynildsrud et al., 2014; Ezewudo et al., 2015; Joseph et al., 2015; Castillo-Ramirez et al., 2016). It has also been employed to study the impact of recombination on bacterial clones (Castillo-Ramirez et al., 2011, 2012) and even to analyse the intra-host evolution of several human pathogens (Diaz Caballero et al., 2015; Azarian et al., 2016). Although pan-genome analyses have been used to indirectly study gene content variation in bacteria, these studies have rarely been used to study gene turnover over very short periods of time and, more importantly, explicitly taking into account the population structure of the bacteria involved. Furthermore, only a few studies have tried to compare the genetic variation generated by point mutation vs. that generated by gene gains and losses within a well-defined population.

We now know much about the microevolution of a few pathogens, such as *Neisseria gonorrhea* or the species from the genus *Chlamydia* (Ezewudo et al., 2015; Joseph et al., 2015), however for most bacterial species we do not know very much. While *Staphylococcus aureus* has been extensively studied (Holden et al., 2010; Nubel et al., 2010; Castillo-Ramirez et al., 2011, 2012), much less is known about the evolutionary dynamics of *A. baumannii*, especially in developing countries (Castro-Jaimes et al., 2016; Grana-Miraglia et al., 2016; Silva et al., 2016; Zenati et al., 2016). Importantly, this bacterial species has emerged as one of the main causes of nosocomial infections over the last decades (Zarrilli et al., 2013; Antunes et al., 2014). One of the factors contributing to this pattern is the ability of this species to show multi-drug (MDR) and extreme (XDR) phenotypes; these phenotypes have been on the rise the last decade. Remarkably, the infections caused by MDR and/or XDR isolates have been linked with higher mortality rates and longer hospitalization (Sunenshine et al., 2007; Metan et al., 2009).

We propose that *A. baumannii* is a good model to study the microevolutionary dynamics of gene content variation for several reasons. First, given that this is a hospital-associated pathogen, it should be rather easy to define single populations. Secondly, considering the highly dynamic genome of this species (Chan et al., 2015), one would expect that gene content should be an important factor as far as the genetic variation is concerned. Here, we use genome sequencing to characterize the microevolution of a single lineage (Sequence Type [ST] 758) within a single locale—a tertiary hospital in a developing country (Mexico). To gain further insights into the evolution of this lineage, we have also incorporated publicly available sequenced strains of this species to create one of the most inclusive data sets to date with 85 genomes and representing 38 STs. We found that gene content variation is of chief importance to generate genetic variation within the population, as it occurs much faster than *de novo* mutations. Furthermore, we also note that more genes are found in the external branches of the phylogeny, which is consistent with the slightly deleterious nature of gene acquisitions.

## Results

### A very recent expanding population

**Figure 1:**
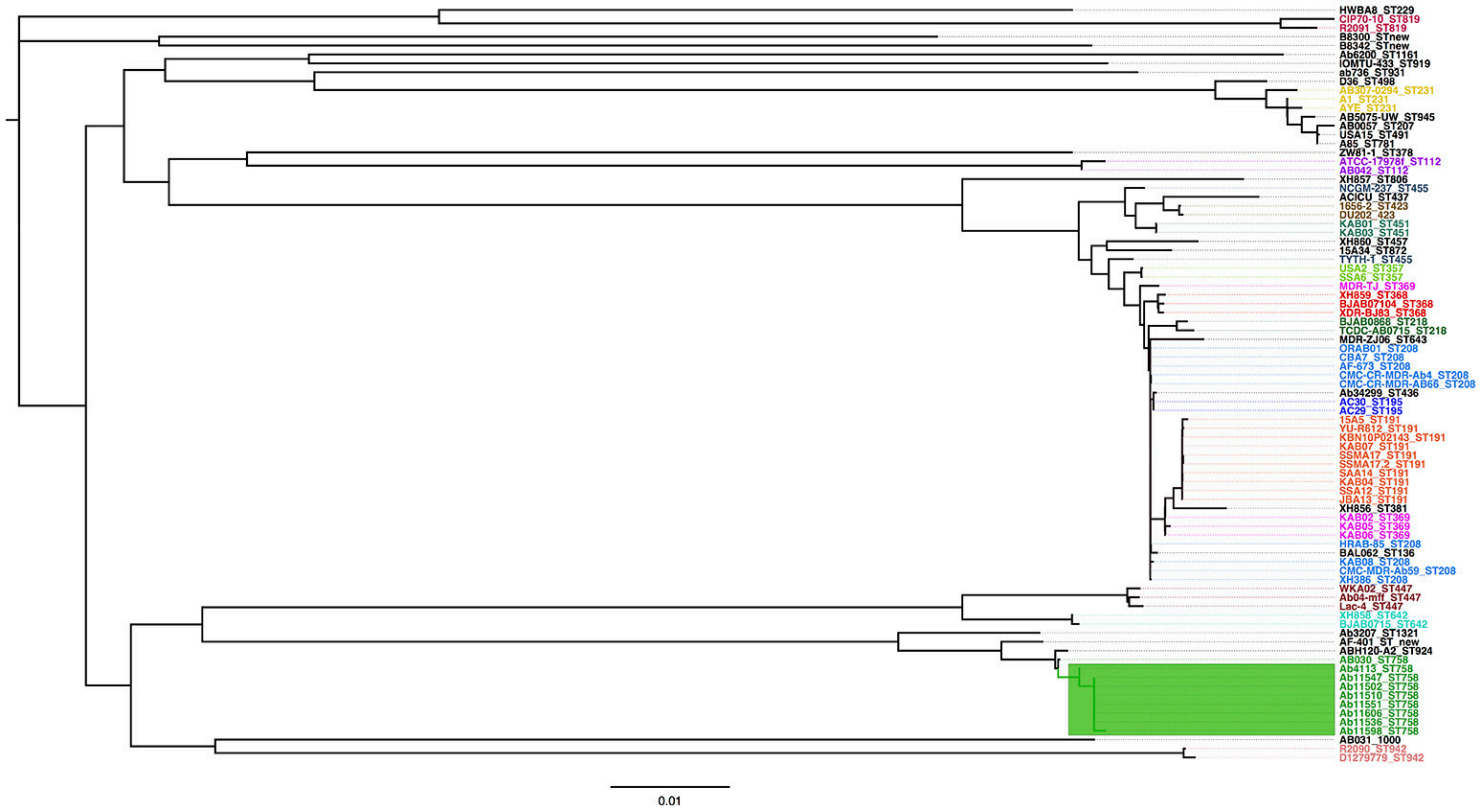
Maximum likelihood phylogeny and population structure analysis. The mid-point rooted phylogeny is based on the concatenated alignment of all the single gene families not affected by recombination and was constructed via PhyML. The color labels represent all the known STs for which there were two or more strains. The green rectangle shows the newly sequenced Mexican strains, which form a single cluster at deepest level of the hierarchical population structure analysis. The scale bar represents substitution per sites.

In order to have a proper data set we first conduct genome sequencing of eight Mexican isolates (all recovered from the same tertiary hospital in Mexico city during 2011-2013, see Supplementary Table 1). Of note, all these isolates belong to the same Sequence Type (ST758). This set was supplemented with a global data set of publicly available genomes (see Supplementary Table 2). Our combined data set has 85 genomes and it is one of the most comprehensive collections in terms of *A. baumannii* lineages (38 STs). Using the combined data set, we initially conducted a Maximum Likelihood (ML) phylogeny on a concatenated alignment of single gene families not affected by recombination to see how the newly sequenced Mexican strains relate to the rest of *A. baumannii* strains. The phylogenetic tree reveals that the Mexican isolates form a tight monophyletic cluster (see green square, Figure 1) and that these are well-differentiated from the remaining strains. A hierarchical population structure analysis at the deepest level (see methods for details) further reinforces this picture, as one of the 32 clusters recovered (green square, Figure 1) has all the newly sequenced Mexican isolates. The very short branches of the Mexican isolates suggest that this population has low genetic variation, to corroborate this we computed the nucleotide diversity for the Mexican isolates and noted that the value is very low (π = 0.000532). Furthermore, there were only 147 segregating sites in this population. Among other things, the low genetic variation in this population may point to a recent introduction of this lineage in to the Mexican hospital. To prove this we conducted a molecular dating analysis (see methods). Our molecular dating analysis confirms this as the time to the most recent common ancestor was found to be mid 2009 (see Supplementary Figure 1). This is consistent with the collection dates of all the Mexican isolates between mid 2011 and late 2013 (see Supplementary Table 1). From Figure 1 is very difficult to appreciate how the Mexican isolates relate to one and another; hence, to examine this in more detail, we constructed a ML phylogeny just considering the Mexican isolates (see Supplementary Figure 2). This phylogeny has a star like topology, which suggests a recent and rapid expansion of this Mexican lineage. To corroborate these findings, we used an independent line of evidence: a Tajima's D analysis on the alignment just containing the Mexican isolates. The clear negative value of Tajima's D, –1.922, implies an overabundance of low frequency polymorphisms and this is consistent with a population size expansion as suggested by the ML phylogeny. Taken together, these results suggest that the Mexican isolates constitute a very young population, which was very recently introduced in Mexico's National Institute of Oncology, and since then has been undergoing a clonal expansion.

It is worth paying attention to the fact that two strains (Ab4113 and Ab11598) from the star like phylogeny of the Mexican lineage show two very long branches, having each one an average distance of over 70 SNPs to the rest of the Mexican strains (see Supplementary Figure 2). We carried out a Tajima's relative test to establish whether those two strains have accumulated significantly more SNPs than the other Mexican strains; this was clearly the case as for both strains, Ab4113 and Ab11598, the null hypothesis of equal rates was rejected when compared to the other Mexican strains. Furthermore, we determined that substitutions defining those two branches were not clustered in a very short region, as might be the case of an unidentified recombination event. Therefore, these two strains may be hypermutator strains, which is in accordance with two recent reports that have found hypermutator strains in *A. baumannii* (Hammerstrom et al., 2015; Komp Lindgren et al., 2016). However, we acknowledge that further experiments are required to better characterize the hypermutator strains found here and we plan to conduct this characterization in the near future.

### High variation in gene content and its dynamics at different scales

**Figure 2:**
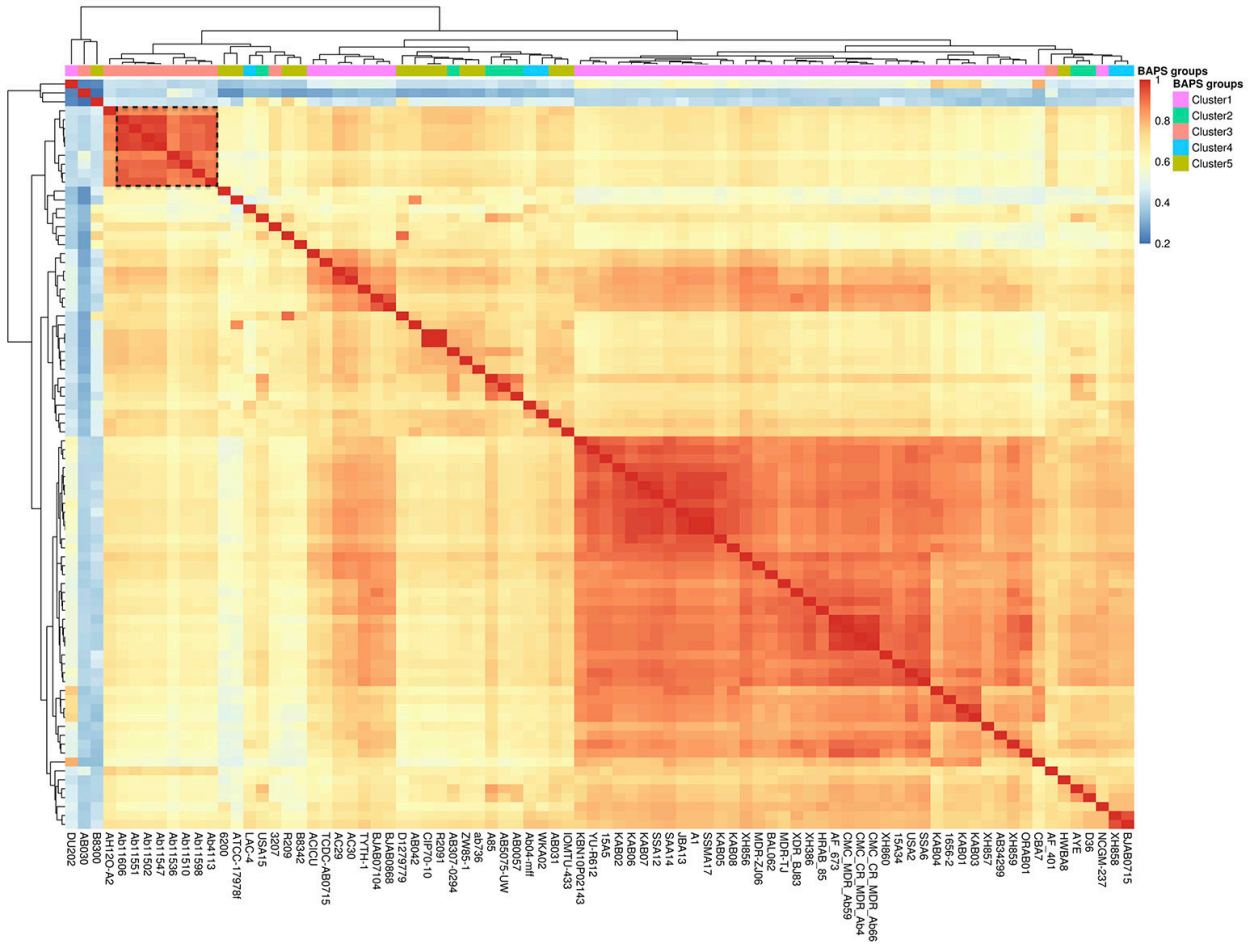
Gene content variation among the strains. Heat map of the gene content correlation matrix used to analyse the gene content differences among the strains. The top row on the heat map shows the BAPS groups of the first level of clustering of the population structure analysis, most of which are split according to the clustering of the heat map. The black dotted rectangle shows the Mexican strains sequenced for this study. The dendrograms across the top and side reflect the clustering by gene content.

Once we had characterized the Mexican population, we wanted to analyse the evolutionary dynamics of gene content variation within it. Here when we refer to gene content variation we mean differences in the number of genes among strains and to measure this we employed closely related homologous groups (CRHGs, see methods). First, to investigate the amount of gene content similarity between the strains, we constructed a gene content correlation matrix and visualized it using a heat map (see Figure 2). It became apparent that there was a considerable amount of variation in gene content among the strains. Furthermore, we observed that the clustering shown in the heat map does not correlate with the groups found in the first level of clustering of the population structure analysis (see BAPS clusters, top row, Figure 2). In other words, the clustering recovered by the heat map did not reflect the grouping in the ML phylogeny and the population structure analysis. However, the newly sequenced Mexican strains (black dotted rectangle in the heat map) appear to be the exception to this, as the clustering shown by these strains is very similar to that of the ML phylogeny. To further elucidate this, we used a distance gene content matrix to construct two Neighbor-joining (NJ) phylogenies based on gene content, one only included the Mexican population and the other involved the whole data set. Next, we tested whether the topologies of these NJ phylogenies were similar to the topology of the ML phylogeny (see Table 1). When the whole data set was included, we did find significant differences between the NJ topology and the topology of ML phylogeny (see Table 1), implying that the phylogenetic relationships inferred from the ML phylogeny were considerably different from the clustering patterns obtained from the gene content variation. However, no significant difference was found when only the Mexican population was considered. Hence, we assume that the most likely explanation is that due to the short time considered, it appeared that the phylogenetic relationships were similar to the clustering patterns gathered from the gene content variation. In summary, the heat map and the topology tests indicate that there is considerable variation in gene content and that only at very short time scales this variation reflects the phylogenetic relationships inferred by the ML phylogeny.

**Table 1:** Topology tests.

	^*^Diff −ln L	**pKH^+^**	**pSH^+^**	**pRELL^+^**
NJ whole data set	−658053.472	0.000	0.000	0.000
NJ Mexican clade	−0.172	0.336	0.352	0.29

*Kishino-Hasegawa (KH), Shimodaira-Hasewaga (SH), and RELL bootstrap proportion tests to determine whether the NJ topologies, based on the gene content matrix, differ significantly from the ML phylogenies*.

* *Difference in log-likelihood to the ML phylogeny*.

+ *p-values under the different tests*.

**Figure 3:**
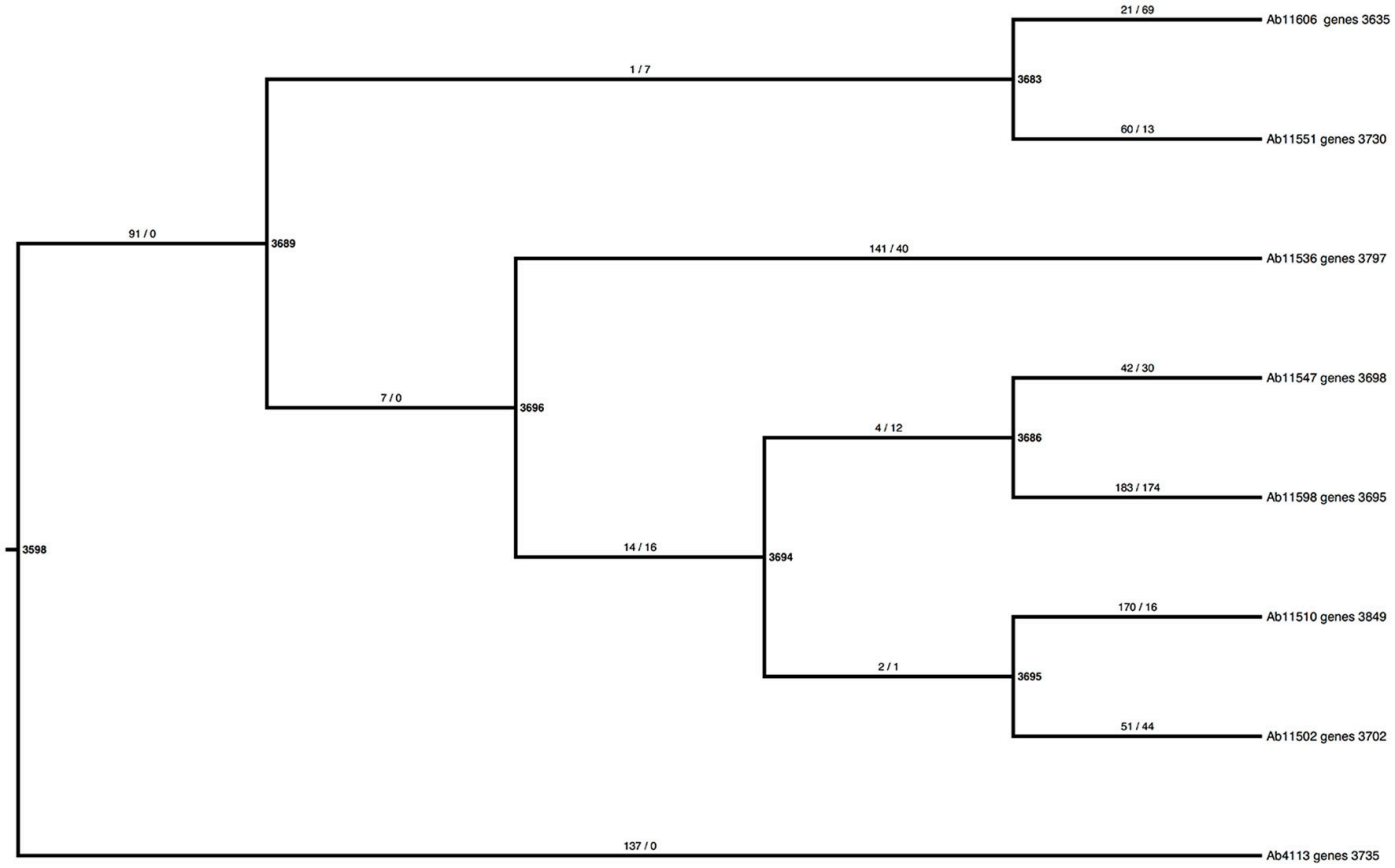
Estimates of the ancestral gene content and the minimum number of losses and gains per branch. The bold numbers next to the nodes show the estimates of the ancestral gene content, whereas the taxa labels give the number of the total gene content for the newly sequenced Mexican strains. The numbers on the branches mark the minimum number of gains (number before the slash) and the minimum number of loses (number after the slash).

To explore the gene content variation dynamics in more detail, we contrasted two evolutionary models of gain and loss of genes across the phylogeny using a probabilistic approach, implemented through BadiRate (see methods). The first model, the global rates model (GD-GR-ML in Table 2), assumes that both the gain and death turnover rates remain constant over time. The second model, the free-rates model (GD-FR-ML, in Table 2) assumes different rates for each branch in the tree. The free-rates model is a better explanation of our data based on the considerable lower value of the Log-Likelihood and, furthermore, as it shows the lowest Akaike Information Criterion (see Table 2). In order to further look into this we also estimated the ancestral gene content and the minimum number of gains/losses in each internal node of the phylogeny again by means of BadiRate. The results are shown in Figure 3. This analysis suggests that there are more genes toward the present than in the past. For instance, the most basal node is the one that shows the least number of genes, whereas the external nodes have many more genes. To establish this more formally, we compared the ancestral gene content of the internal branches of the phylogeny vs. the gene content of the external branches. We noted that considerably more genes are present in the external branches (mean = 3,770) than at the internal nodes (mean = 3,677, Wilcoxon rank sum test W = 7.5, *p*-value = 0.02052). This supports the idea that the presence of fewer genes in the internal branches might be due to the selective removal of genes, which could have deleterious effects. We think that given sufficient time purifying selection will remove most of the genes but at the tips of the tree there has not been enough time for that to happen. To sum up this part, this array of analyses shows that gain and loss of genes do not remain constant over time and there seems to be more genes toward the present time and this could be due to natural selection (purifying selection).

**Table 2:** Branch models of gene family turnover.

**Branch model**	**−lnL^+^**	K^*^	**AIC**	**ΔAIC^&^**
GD-FR-ML	−7500.2956	29	15058.59	0.00
GD-GR-ML	−8290.2042	3	16586.41	15015.68

*The GD-GR-ML model implies that all the branches have the same turnover rates, whereas GD-FR-ML model assumes that each branch has its own turnover rates. In both models, turnover rates were estimated by Maximum Likelihood (ML)*.

+ Log-Likelihood scores;

* Number of parameters;

& *ΔAIC is the difference in the Akaike Information Criterion (AIC) for each model to the best model. We used BadiRate to implement the Gain-and-Death stochastic population model to estimate the gene family turnover rates*.

### Comparison of gene turnover vs. accumulation of *de novo* mutation

**Figure 4:**
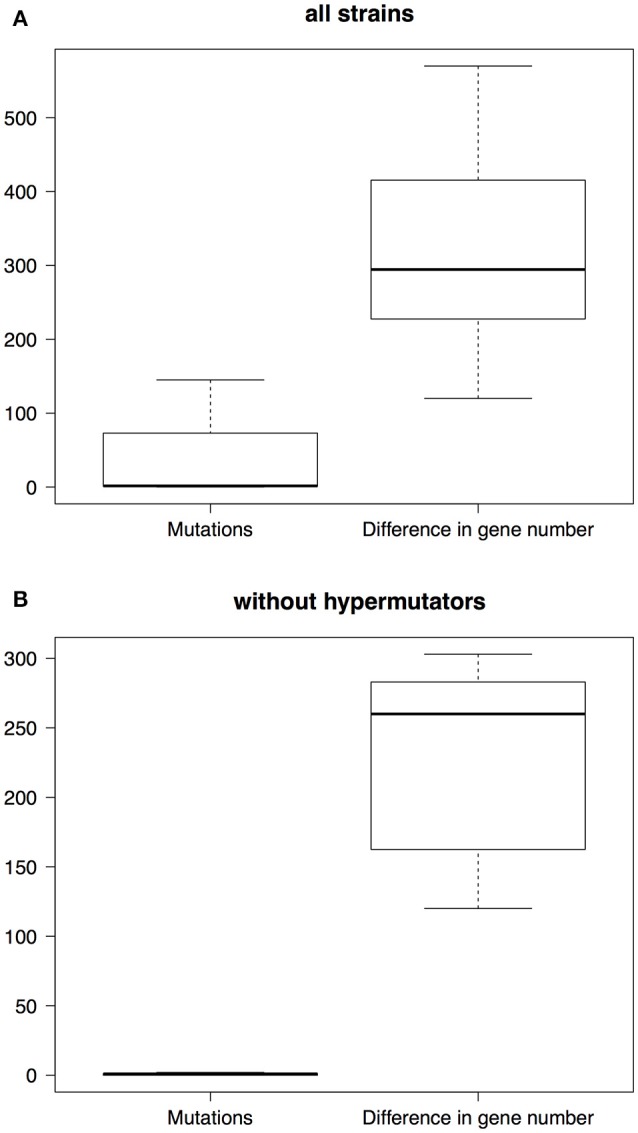
Boxplots of the differences due to either mutations or gene losses/gains. The boxes on the left refer to the differences due to mutations, whereas the boxes on the right describe the differences in gene content. We carried out pairwise comparisons including all the strains **(A)** and pairwise comparisons without the hypermutator strains **(B)**.

Thus far we have analyzed the dynamics of gene turnover but we have not compared this to a more typical source of variation, i.e., mutation. Given the very recent emergence of the Mexican population it is a suitable scenario to compare the contribution of gene content variation and the accrual of *de novo* mutations to genetic variation. We carried out pairwise comparisons of Mexican strains as a means to conduct such analysis. On the one hand, we computed the number of mutations that differentiate any two Mexican strains and, on the other hand, we calculated the number of genes in which any two Mexican strains differ. Most of the strains differ in just a few mutations from one another (boxplots on the left, Figure 4), the median difference being just 1.5 mutations. There were six pairwise comparisons that involved strains that did not have any mutations to differentiate them. However, these strains differed from each other by hundreds of genes (boxplots on the right, Figure 4), with a median value of 294.5 genes and, remarkably, no two strains were equal to each other in terms of their gene content. Irrespective of the inclusion of hypermutator strains (see Discussion) or not, this analysis clearly showed that the rate of gene turnover is much higher than the accumulation of mutations (see Figure 4); this difference was statistically significant either way (Wilcoxon rank sum test with hypermutators, *p*-value = 1.573e-10 and without hypermutators, *p*-value = 2.498e-06). For instance, if the hypermutators are included in the comparisons the mean number of mutations is 36.75, whereas the mean number in gene differences is 314.2; this is also evident when hypermutators are left out, as the mean number of mutations is 0.6667, whereas the mean number of gene differences is 224. However, the difference in gene content between any two strains might have been due to a small number of events—just because several genes can be introduced at once thanks to Mobile Genetic Elements (MGEs), for example Insertion Sequences (ISs) or phage. In order to explore this, we compared four sets of two strains that both were sampled the same year (see Supplementary Table 4); we assumed that two or more genes were introduced in the same event if they are contiguous to one another. In all the four pairwise comparisons the number of events is considerably lower than the number of gene differences. For instance, in the first comparison (involving Ab11502 and Ab11510), although these two strains differ in 311 genes (82 present in Ab11502 but not in Ab11510 and 229 in Ab11510 but absent in Ab11502), we estimated that around 112 events have produced this difference in genes (72 events in Ab11510 and 40 in Ab11502). Clearly, the same pattern applies to the rest of the comparisons and implies that several genes are introduced/lost simultaneously (see Supplementary Table 4).

Notably, many of these genes, ~52% (see Supplementary Table 4 for the value for each comparison), could be associated with MGEs, such as ISs, phage or plasmids. Along these lines, we found that the accessory genome of the Mexican strains is enriched in MGEs (Chi-squared test, *p*-value < 2.2e-16) compared to the core genome. Finally, it is worth mentioning that this gene turnover has affected both plasmids and chromosomes and it is not only due to the loss and gain of plasmids. First, we noted that a significant number (more than 60% in all comparisons) of the unique genes in the pairwise comparisons have significant hits in chromosomes from complete genome sequences (see third column, Supplementary Table 4). Second, we conducted a plasmid profile analysis of the Mexican strains (see Supplementary Figure 3) and noted that six of the eight strains had identical plasmid profiles, which suggests that the difference in gene content is not due to the loss or gain of complete plasmids for these six strains. Whereas, the two remaining strains have the same set of bands as the other six strains plus 1 and 3 extra bands. These last two analyses imply that gene content variation cannot be exclusively due to gain and loss of plasmids and that this variation has considerably affected the chromosome. Collectively, these data indicate that gene turnover (mediated by MGEs) has happened considerably more rapid than the accumulation of mutations and that this rapid turnover is an important force in generating genomic variation in both plasmid and chromosomes.

## Discussion

In this study we used a population-genomics approach to analyse the evolutionary dynamics of gene content over a very short period of time and from a single population. In order to do this, we sampled several strains (all from the same lineage) from a tertiary hospital in a developing country (Mexico). To put this lineage in the context of the global population of *A. baumannii*, we have also incorporated publicly available genomes to create one of the most inclusive data sets for this bacterium, as far as different lineages (STs) are concerned—more than 35 STs are represented in our data set. All the newly sequenced Mexican strains belong to the ST758 and, notably, the PubMLST webpage (https://pubmlst.org) only reports two other strains with this ST. One is the Canadian strain AB030, which was included in our data set, and the other is a Mexican strain, isolate 6463. Thus far, this ST has mainly been reported in North America and it might be that this ST is endemic in this region; however a recent report found this ST also in a hospital in Pretoria, South Africa (Lowings et al., 2015). Many more isolates from different parts of the world are needed to properly establish the distribution of this ST; nonetheless this ST belongs to the clonal complex 636 (as per the Oxford MLST scheme), which is not within the global clones (GCs), and, therefore, it is very unlikely that ST758 would be as widely distributed as STs within the GCs. With respect to the *A. baumannii* populations in Mexico, we are adamant that our study could be of paramount importance for future studies tackling the population genomics of this species in this country. Clearly this is not the first study analyzing *A. baumannii* isolates from Mexico; in fact, over the last decade there have been several studies (Ares et al., 2013; Morfin-Otero et al., 2013; Alcantar-Curiel et al., 2014; Bocanegra-Ibarias et al., 2015; Cornejo-Juarez et al., 2015; Gonzalez-Villoria et al., 2016; Tamayo-Legorreta et al., 2016), which have been very useful from a clinical point of view, as they focused on the molecular epidemiology (Morfin-Otero et al., 2013; Gonzalez-Villoria et al., 2016; Tamayo-Legorreta et al., 2016) or the antibiotic resistance profiles of hospital isolates (Ares et al., 2013; Alcantar-Curiel et al., 2014; Bocanegra-Ibarias et al., 2015). However, none of those studies have analyzed the genomic diversity of the isolates in question and, to the best of our knowledge, this is the first study that has addressed the genetic variation within a population of *A. baumannii* isolates in Mexico at a genome level. Our phylogenetic and molecular dating analyses indicated that the Mexican strains comprise a very recently founded population undergoing a rapid expansion. Notably, similar rapid clonal expansions have been also described for some STs of *S. aureus* (Aanensen et al., 2016) and, very likely, this is a common trend exhibited by many bacterial populations within the hospital setting.

Although pan-genome analyses have been extremely useful, it should be remembered that they could also be misleading. First, core and accessory genomes are bound to be artifacts of the sampling criteria employed and, importantly, the genes falling in each of these two categories totally depend on the taxonomic level chosen. Here, we avoided these distinctions and we considered all the genes for the gene content analysis, whereas for defining the global population and the Mexican clade we used single gene families (without signals of recombination) as a proxy for orthologous genes. Therefore, our strategy considerably improves on previous studies that have used pan-genome analyses.

Although there have been some studies that have gone beyond the use of pan genome analysis, and have even used proper statistical approaches, these have considered broader timescales and did not take the population structure into account (Librado et al., 2014; Nowell et al., 2014). Furthermore, none of those studies compared the rate of change due to gene content variability to that attributable to *de novo* mutation. To the best of our knowledge, this is the first study that not only takes into account the population structure to proper understand gene content variation among strains but also compares this gene content variability to a better known source of genetic variation, that is mutation. However, we note that similar trends have been very recently described in a work studying within-patient genomic diversity of *Vibrio cholera* isolates (Levade et al., 2017).

In this study, we were not only able to show that rapid gene turnover has been a major factor in the generation of genomic variation in the few years of existence of this expanding Mexican population but also to show that, on average, gene turnover has introduced more than 8 times more variation than mutation and this is taking into account the hypermutator strains. However, if hypermutator strains are not included in the analysis, on average, gene turnover has added ~335 more variation than mutation. Notably many of these gene differences are not independent, as the genes were introduced in the same event via some MGE; in conformity with this, we found that the accessory genome is enriched in MGEs. Nonetheless, chromosome regions were also implicated in this type of variation. Notably at this short time scale gene content variation still reflects the common ancestry relationships of the strains (i.e., the topology of the ML likelihood) and, given the increased resolution, gene content variability could be very useful for studying outbreaks or other events that happen at very short periods of time.

We reason that patterns found in the ancestral gene content (external branches showing more genes) are compatible with the slightly deleterious nature of many gene acquisitions. Although some gene acquisitions might be beneficial initially, most of them have a fitness cost and therefore will be eliminated by natural selection. Nevertheless, if the fitness cost of these acquisitions is not high (i.e., slightly deleterious) the removal process will not be happening instantaneously—giving time for acquisitions to be sampled if the sampling strategy considers very closely related isolates. Under this scenario, external branches would be enriched in gene acquisitions, as selection has not had enough time to remove them; whereas, internal branches would have less genes, as natural selection has had more time to purge those slightly deleterious acquisitions. Furthermore, the slightly deleterious nature of the gene content variability is further reinforced by the fact that the global rates model (GD-FR-ML in Table 2) assuming different gain and death turnover rates over time was a considerably better fit for our data. Interestingly, Wolf and Koonin (2013) have discussed the idea that genome reduction—either by neutral or selective processes—is the principal mode of evolution, although their arguments were based on studies that had considered macro evolutionary scales rather than micro evolutionary scales. In a slightly different view, Gogarten and Townsend have also discussed that most of the genes horizontally transferred have neutral or nearly neutral effects in the receiver genome and just a few of those genes have a positive effect (increase the fitness) in the recipient genome (Gogarten and Townsend, 2005). Along these lines, several studies have proposed that many gene gains/losses have minor effects on the fitness of bacteria and behave rather neutral (Baumdicker et al., 2012; Knoppel et al., 2014; Andreani et al., 2017).

In conclusion, our study shows that gene content variability could be a major source of genetic variation that takes place much faster than the accumulation of *de novo* mutations in bacterial populations at very early stages of diversification. Furthermore, it suggests that the loss of genes in the internal branches is due to the gradual removal of the slightly deleterious gene acquisitions. This study clearly demonstrates the utility of NGS for studying the micro evolutionary dynamics of gene content variation over very short periods of time and for comparing this to more typical forms of genetic variation such as mutations.

## Materials and methods

### Genomes used and homologous groups

Because we wanted to know the evolutionary dynamics of a single lineage in a single location over a very short period of time, we selected eight *A. baumannii* isolates (Supplementary Table 1), all of which belong to ST758 (according to the Oxford MLST scheme), from the Instituto Nacional de Cancerología (Mexico's National Institute of Oncology) that is a tertiary hospital located in Mexico City. The antibiograms and the source of the Mexican isolates are provided in Supplementary Table 3. This study was carried out with isolated strains, confidentiality of the patients is preserved and there is no possible way to link the information here provided to any individual; thus, the Ethics Committee approval of the Instituto Nacional de Cancerología was not required. All the isolates were sequenced by means of an Illumina MiSeq platform, considering 250-bp paired-end reads. We used the SolexQA software (Cox et al., 2010) to trim the reads prior to assemble the genomes. These draft genomes were assembled using Velvet version 1.2.09 (Zerbino and Birney, 2008) and Spades v3.9.0 (Bankevich et al., 2012) and contigs smaller than 300 base pairs were not taken into account. We manually edited our assembly for gap closure and error correction. The whole genome sequences have been deposited at DDBJ/ENA/GenBank under the accession numbers MSCX00000000, MSCY00000000, MSCZ00000000, MSDA00000000, MSDB00000000, MSDC00000000, MSDD00000000 (BioProject PRJNA355850). One of the isolates (Ab11510) was also sequenced using Pacific Biosciences technology and assembled with SMRT (accession numbers CP018861, CP018862 and CP023300). Although the quality of these assemblies varied to some extent, all these draft genomes have good values considering the coverage (range 49.5–375) and the number of contigs was below 90 in all cases (Supplementary Table 1). We annotated these Mexican isolates through PROKKA v1.11 (Seemann, 2014). We also included 77 *A. baumannii* complete genomes from the NCBI (see Supplementary Table 2); these include isolates from Europe, Asia, South and North America. For consistency, we also annotated these genomes using PROKKA v1.11 (Seemann, 2014). Then, in order to construct the homologous groups, we used the program PanOCT (Fouts et al., 2012). We first carried out BLAST searches between all genomes, considering an e-value of 1.0 e^−30^, we then fed these to PanOCT to be able to create the homologous groups. Because we wanted to define closely related homologous groups (CRHGs), we required that sequences aligned ≥90% of their lengths and were ≥80% identical when running PanOCT—other than that, default parameters were used. We identified a total of 14194 homologous groups, of which 1462 belong to the core genome according to PanOCT. We identified 1383 single gene families, homologous groups that have only one gene per genome. We also assigned, when possible, the CRHGs to their potential MGEs, for doing that we used two databases: ISfinder (Siguier et al., 2006) and ACLAME (Leplae et al., 2010). We conducted BLAST searches for each gene of each CRHG against those two databases with an e-value of 1.0 e^−30^ and requiring that the query sequence aligned ≥60% of its length and was ≥40% identical. Finally, we also ran another pangenome analysis, again via PanOCT, but this time just considering the Mexican strains.

### Phylogenies, molecular dating, and population genetics analysis

Identifying true orthologous genes is not trivial (Castillo-Ramirez and Gonzalez, 2008), however as proxy for orthologous genes we used single gene families. We employed a concatenated alignment of 1383 single gene families to conduct a mixture analysis via the Bayesian Analysis of Population Structure Analysis (BAPS) program version 6 (Tang et al., 2009). We ran a hierarchical model-based clustering of the strains implementing the tandem version of BAPS (Cheng et al., 2013). This clustering was carried out setting four levels in the hierarchy. To get a measure of the number of genetically diverged groups we conduct a preliminary analysis setting the maximum number of genetically differentiated groups to 35, as it has been done previously for other bacterial species (Joseph et al., 2015), and to 35 in the final analysis. The first level of clustering yielded five clusters, the second produced 15, the third provided 22, and the last one gave 32. We constructed a species phylogeny for this data set using a concatenated alignment of 574 single gene families that did not show evidence of recombination, as inferred by the test for detecting recombination implemented via the PhiPack program (Bruen et al., 2006). We ran a Maximum Likelihood phylogeny on the concatenated alignment through PhyML (Guindon et al., 2010) and setting the model described below. We conducted statistical model selection, as in Lopez-Leal et al. (2016), to find the most adequate model. This analysis was done by means of jModelTest (Abascal et al., 2005) and the model selected was GTR+R+I. We also made another phylogeny just considering the Mexican isolates also via PhyML. We created a SNPs alignment, from the concatenated alignment of the 574 single genes families not affected by recombination by keeping only the variable sites. We used the SNPs alignment to carry out a dating analysis, which was conducted via BEAST2 (Bouckaert et al., 2014). For the SNP alignment only a subset of strains, for which we had reliable information on the date of isolation, were considered (see Supplementary Table 6) so to be able to calibrate the relaxed molecular clock confidently. We set a log-normal relaxed clock, employing the GTR DNA model and using the correction for among site variation; this model was chosen as the statistical model selection analysis, implemented via jModelTest2 (Posada, 2009; Darriba et al., 2012), indicated that was the most suitable model for this data set. This analysis was run for 200,000,000 generations, sampling every 10,000 generations and discarding the first 20,000,000 generations as burn-in. We used VariScan version 2.0 (Vilella et al., 2005) to compute some population genetics summary statistics (nucleotide diversity [π] and Tajima's D) setting the runmode to 12. We employed MEGA6 (Tamura et al., 2013) to carry out Tajima's relative test and evaluate whether the potential hypermutators strains have higher evolutionary rates. We conducted two analyses: one considering the strain Ab4113 and other involving the strain Ab11598. In both analyses, the out group was Ab11551 and the taxon B was Ab11510.

### Gene content analysis and pairwise comparisons

We have created a matrix that contains the number of genes per homologous groups and the genomes considered. We normalized the matrix by dividing each value by the sum of all the values. Using this matrix we have created two additional matrices used for downstream analysis. One was a correlation matrix obtained using the cor() function in R and setting a Pearson correlation. The correlation matrix was visualized using a heat map employing the pheatmap() function also in R. We also constructed a distance matrix, for which we utilize the dist() function and the distance measure used was “Euclidean.” Then, by means of the APE library in R (Paradis et al., 2004) we used the Neighbor-joining algorithm to construct two phylogenies based on the distance matrix. We conducted several topology tests (see Table 1) to determine whether the NJ phylogenies based on the gene content matrix differ significantly from the ML phylogenies; the topology tests were implemented via PAML 4 (Yang, 2007). We implemented a gain-death model stochastic by means of the BadiRate software (Librado et al., 2012). This analysis requires an ultrametric tree of the taxa considered, therefore we used the APE library (Paradis et al., 2004) to convert the ML phylogeny of the Mexican clade into an ultrametric tree. We considered two branch models: one was the global rates model (GD-GR-ML) and the other was the free-rates model (GD-FR-ML). To assess the goodness of fit of these two models we used the log-likehood of the models and the Akaike Information Criterion (Akaike, 1974). We also set the option “anc” that gives the number of genes at the internal branches for each gene family and the total number of genes; it also reports the minimum number of losses and gains per internal and external branch. We also carried out a set of pairwise comparisons (see Supplementary Table 4); for each comparison we determine the number of genes present in one but not the other strain and the sum of those unique genes per strain gives the total difference in gene number between the two strains under consideration. We assigned a potential chromosomal location for the unique genes per strain using a set of chromosomes from complete genomes, which are listed in Supplementary Table 5. For doing this, we carried out BLAST searches for each gene present in one but not the other strain against a database containing the chromosomes mentioned above with an e-value of 1.0 e^−30^ and requiring that the query sequence aligned ≥60% of its length and was ≥40% identical.

## Author contributions

SC conceived, designed, and coordinated the study. LG assembled and annotated the genomes, constructed the homologous groups, ran the ML phylogeny and created the gene content matrices. SC conducted the population structure and molecular dating analyses, ran the gene turnover analysis, and conducted the population genetic parameters. LL helped with the genome assemblies and genome annotations. ÁP conducted the plasmid profiles. PV, CV, and MC contributed the Mexican isolates and participated in the general discussion. SC and LG wrote the manuscript. All the authors revised and approved the manuscript.

### Conflict of interest statement

The authors declare that the research was conducted in the absence of any commercial or financial relationships that could be construed as a potential conflict of interest.

## Supplementary material

Supplementary Table 1 Newly sequenced Mexican isolates on this study.

Supplementary Table 2 Publically available complete genomes downloaded from the NCBI.

Supplementary Table 3 Antibiograms and source of the Mexican isolates.

Supplementary Table 4 Pairwise comparisons of a set of strains.

Supplementary Table 5 List of the complete genomes from which chromosomes were extracted to assign potential chromosomal localization for the gene differences regarding the pairwise comparisons of the set of strains used in Supplementary Table 4.

Supplementary Table 6 Strains, and their dates of isolation, used for the molecular dating analysis.

Supplementary Figure 1 Molecular dating Mexican clade. Marginal posterior distribution for the time to the Most Recent Common Ancestor of the (tMRCA) Mexican strains. Blue bars denote the 95% highest posterior density interval. The mean and median values for the tMRCA are also provided.

Supplementary Figure 2 Phylogeny Mexican clade. The phylogeny is based on the concatenated alignment of all the single gene families not affected by recombination and was constructed via PhyML. This phylogeny includes only the newly Mexican strains. The red labels denote the hypermutators strains, the number on the branches denote the average SNP distance to the non-hypermutators strains. The scale bar represents substitution per sites.

Supplementary Figure 3 Plasmid profile analysis of the Mexican strains. Plasmid profiles of the 8 Mexican isolates analyzed by agarose gel electrophoresis (0.8%). Names of strains are listed at the top the figure. The first and last lanes show the plasmid profile of *Acinetobacter haemolyticus* 11616 that was utilized as molecular weight standard. Arrows at the right indicate the plasmid molecular weights of *A. haemolyticus* 11616 and the migration point of broken DNA.
