# Supplementary material for: Rapid Gene Turnover as a Significant Source of Genetic Variation in a Recently Seeded Population of a Healthcare-Associated Pathogen
Source: Front Microbiol. 2017 Sep 20;8:1817. doi: 10.3389/fmicb.2017.01817 (PMC5611417; doi:10.3389/fmicb.2017.01817)
Supplement: Supplementary Table 4 — Pairwise comparisons of a set of strains. [file Table4.PDF]

Supplementary Table 4

Pairwise comparisons of a set of strains

Pairs of Strains	Uniq Genes	Events	% Genes with Potential Chromosomal Localization	% Genes Potentially Being MGEs
**Ab11510**	229	72	70.74	58.95
**Ab11502**	82	40	59.76	42.68
**Totals**	311	112	67.85	54.66
**Ab11598**	193	120	87.05	46.63
**Ab11551**	215	96	80.00	45.58
**Totals**	408	216	78.92	44.12
**Ab11536**	132	54	60.61	59.85
**Ab11606**	47	27	59.57	59.57
**Totals**	179	81	60.34	59.78
**Ab11547**	137	48	71.53	49.64
**Ab4113**	165	65	52.73	48.48
**Totals**	302	113	61.26	49.01
