# Supplementary material for: Rapid Gene Turnover as a Significant Source of Genetic Variation in a Recently Seeded Population of a Healthcare-Associated Pathogen
Source: Front Microbiol. 2017 Sep 20;8:1817. doi: 10.3389/fmicb.2017.01817 (PMC5611417; doi:10.3389/fmicb.2017.01817)
Supplement: Supplementary Table 5 — List of the complete genomes from which chromosomes were extracted to assign potential chromosomal localization for the gene differences regarding the pairwise comparisons of the set of strains used in Supplementary Table 4. [file Table5.PDF]

List of the complete genomes from which chromosomes were extracted to assign potential chromosomal localization for the gene differences regarding the pairwise comparisons of the set of strains used in supplementary table 4.

ACICU
AYE
AF_401
BJAB07104
Ab3207
D36
KB05
A85
