# Supplementary material for: Rapid Gene Turnover as a Significant Source of Genetic Variation in a Recently Seeded Population of a Healthcare-Associated Pathogen
Source: Front Microbiol. 2017 Sep 20;8:1817. doi: 10.3389/fmicb.2017.01817 (PMC5611417; doi:10.3389/fmicb.2017.01817)
Supplement: Supplementary Table 6 — Strains, and their dates of isolation, used for the molecular dating analysis. [file Table6.PDF]

Strain Name	Isolation Date
ACICU	2005
AB3070294	1994
AB0057	2001
AYE	2001
D1279779	2009
MDR-ZJ06	2006
TYTH-1	2008
BJAB0715	2008
ZW85-1	2013
AC29	2011
AB031	2010
LAC-4	1997
Ab6200	2012
NCGM-237	2012
IOMTU-433	2013
A1	1982
ORAB01	2012
AB5075-UW	2008
XH386	2014
Ab04-mff	2012
R2090	2013
D36	2008
R2091	2014
YU-R612	2014
XH860	2009
XH859	2009
XH857	2010
XH856	2010
XH858	2010
Ab3207	2008
AC30	2011
Ab11536	2011
Ab11598	2011
Ab4113	2013
Ab11547	2013
Ab11551	2011
Ab11502	2012
Ab11606	2011
Ab11510	2012
