# Supplementary figures and images for: Rapid Gene Turnover as a Significant Source of Genetic Variation in a Recently Seeded Population of a Healthcare-Associated Pathogen
Source: Front Microbiol. 2017 Sep 20;8:1817. doi: 10.3389/fmicb.2017.01817 (PMC5611417; doi:10.3389/fmicb.2017.01817)

# time to the Most Recent Common Ancestor (tMRCA) Mexican clade

Mean = 2009.59, Median = 2009.6348

95% HPD Interval [2008.40, 2010.55]

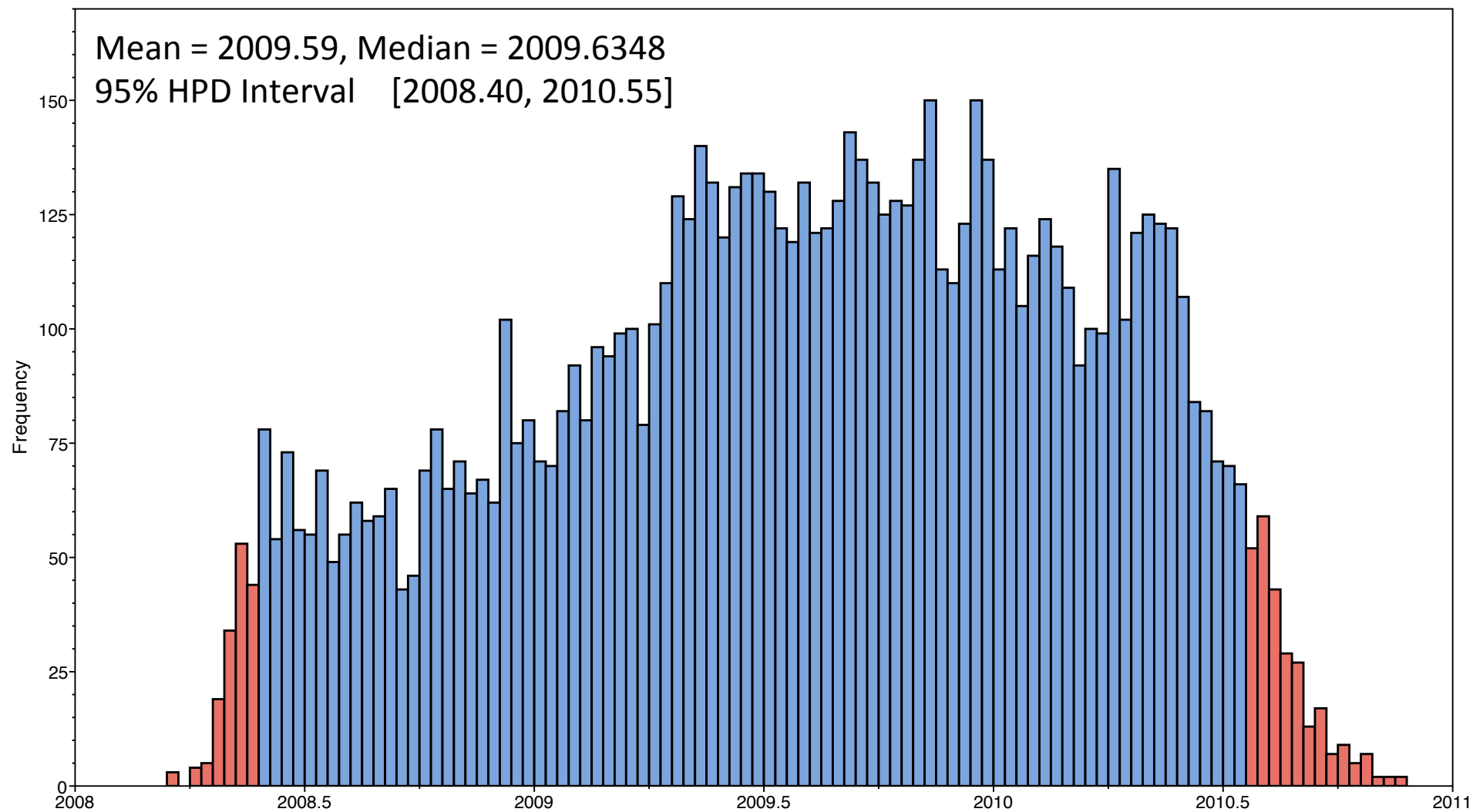

Supplement: Supplementary Figure 1 — Molecular dating Mexican clade. Marginal posterior distribution for the time to the Most Recent Common Ancestor of the (tMRCA) Mexican strains. Blue bars denote the 95% highest posterior density interval. The mean and median values for the tMRCA are also provided. [file Image1.PDF]

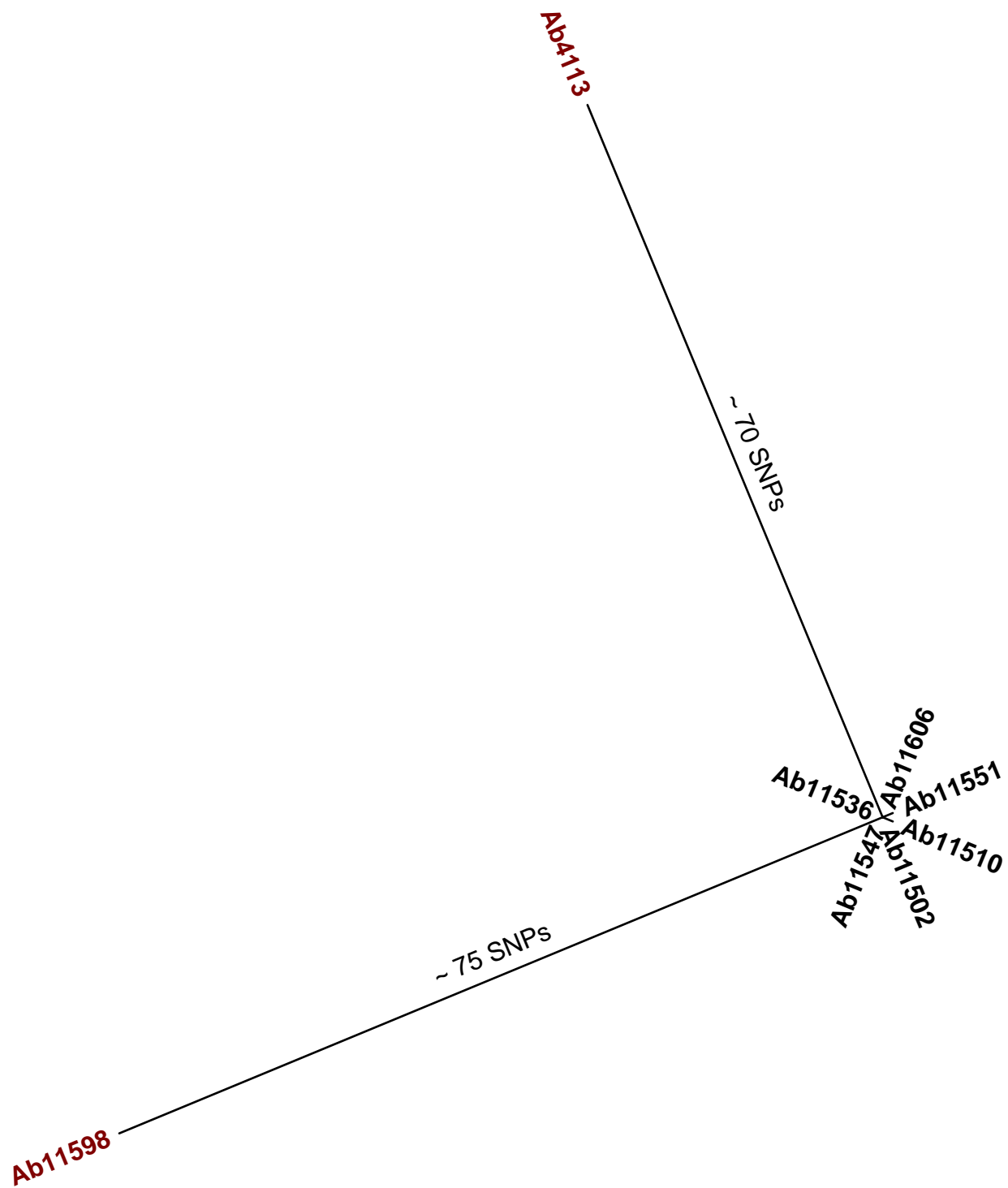

Supplement: Supplementary Figure 2 — Phylogeny Mexican clade. The phylogeny is based on the concatenated alignment of all the single gene families not affected by recombination and was constructed via PhyML. This phylogeny includes only the newly Mexican strains. The red labels denote the hypermutators strains, the number on the branches denote the average SNP distance to the non-hypermutators strains. The scale bar represents substitution per sites. [file Image2.PDF]

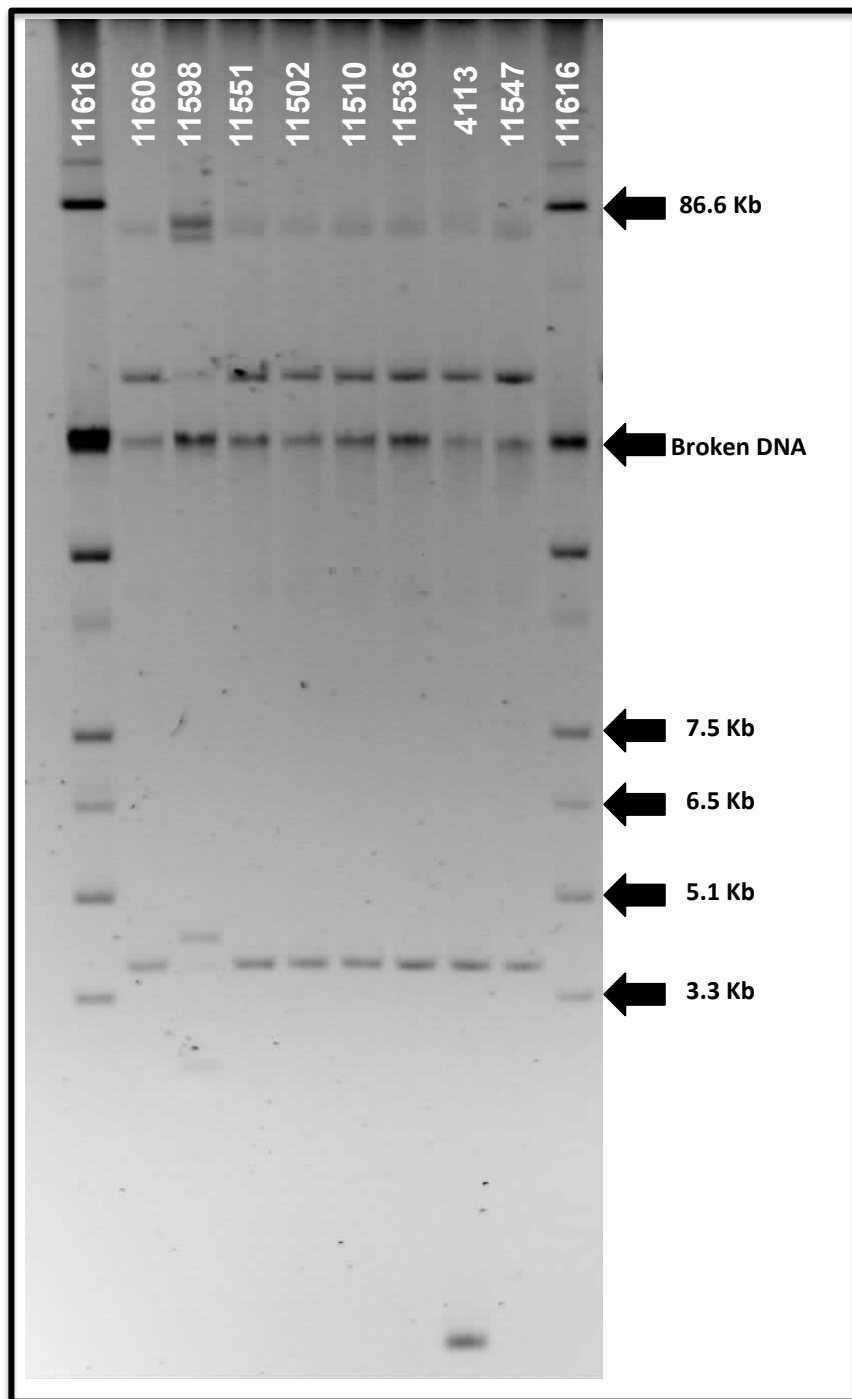

Supplement: Supplementary Figure 3 — Plasmid profile analysis of the Mexican strains. Plasmid profiles of the 8 Mexican isolates analyzed by agarose gel electrophoresis (0.8%). Names of strains are listed at the top the figure. The first and last lanes show the plasmid profile of Acinetobacter haemolyticus 11616 that was utilized as molecular weight standard. Arrows at the right indicate the plasmid molecular weights of A. haemolyticus 11616 and the migration point of broken DNA. [file Image3.PDF]
